# Supplementary material for: Chromosome instability induced by a single defined sister chromatid fusion
Source: Life Sci Alliance. 2020 Oct 26;3(12):e202000911. doi: 10.26508/lsa.202000911 (PMC7652394; doi:10.26508/lsa.202000911)
Supplement: Supplementary file 7 [file LSA-2020-00911_Supplemental_Data_4.pdf]

Supplementary Method

overview

This document is written to provide supplementary information for the paper entitled ‘Chromosome Instability Induced by a Single Defined Sister Chromatid Fusion’ by K. Kagaya, N. Noma-Takayasu, I. Yamamoto, S. Tashiro, F. Ishikawa, M. T. Hayashi. The codes and data are available from the figshare repository (https://doi.org/10.6084/m9.figshare.7929266).

The reasons why we chose the approach (Bayesian model evaluation with WAIC) are three folds:

1. to consider a hierarchical structure (lineage tree),
2. to compare the hierarchical model with non-hierarchical model,
3. to minimize the generalization error (maximum likelihood estimation is worse than Bayes).

The approach is based on the mathematically rigorous theory (Watanabe, 2018, Mathematical Theory of Bayesian Statistics; http://watanabe-www.math.dis.titech.ac.jp/users/swatanab/waicwbic_e.html (http://watanabe-www.math.dis.titech.ac.jp/users/swatanab/waicwbic_e.html)).

The framework and procedure we took here may not be a conventional and familiar one for the biological community. However, our approach is beginning to be accepted in the papers (Wakita, Kagaya and Aonuma, 2020 J Royal Soc Interface; Harada, Hayashi and Kagaya, 2020 PeerJ).

Currently, WAIC is the best estimator of the generalization error of the statistical models. For the non-regular models including the generalized linear models (GLMMs), WAIC is more powerful than AIC based on maximum likelihood method.

AIC and WAIC are the estimators of the generalization error: the discrepancy in the unknown true distribution that generates data q(x) and the predictive distribution that built using a set of realized data p(x|Data) (see the discussion of the paper, Harada, Hayashi and Kagaya, 2020 PeerJ).

To build p(x|Data), AIC is based on the maximum likelihood method. AIC has been often used for GLM. However, if we want to compare GLM with GLMM, WAIC based on the Bayesian method is better than AIC. GLMM has been widely used because it is appropriate for the clustered or grouped data (Lord, et al., 2020; Arts, et al., 2015).

Our live cell data has tree structure, so we took into account the structure in the models and used WAIC to evaluate the models from the predictive point of view (Akaike, 1974; Sakamoto, Ishiguro & Kitagawa, 1986; Watanabe, 2018).

Here we walk through the two parts of the statistical inferences:

1. probability of micronucleus(MN) formation (9 models)

model_name	intercept					WAIC	dWAIC(nat/sample)	dWAIC(bit/sample)	distribution	distribution2
model_1_1r	hierarchical_b	SCF	RNF	SIS2-3	Stage	0.1349479	0.04442622	0.064093485	bernoulli	“normal(b0, bs)”
model_1_2r	b	SCF	RNF	SIS2-4	Stage	0.09097437	0.00045269	0.000653094	bernoulli	
model_1_3r	hierarchical_b	SCF				0.09479229	0.00427061	0.006161188	bernoulli	“normal(b0, bs)”
model_1_4r	b	SCF				0.09052168	0	0	bernoulli	
model_1_5r	hierarchical_b					0.1215877	0.03106602	0.044818792	bernoulli	“normal(b0, bs)”
model_1_6r	b					0.1039501	0.01342842	0.019373114	bernoulli	
model_1_3r_st	hierarchical_b	SCF				0.1029526	0.01243092	0.017934026	bernoulli	“student_t(4, b0, bs)”
model_1_4r_st2	hierarchical_b	SCF				0.1315296	0.04100792	0.059161921	bernoulli	“student_t(2, b0, bs)”
model_1_4r_st3	hierarchical_b	SCF				0.225825	0.13530332	0.195201423	bernoulli	“student_t(1, b0, bs)”

2. impact of the MN formation to the duration of interphase (12 models)

model_name	intercept	WAIC	dWAIC(nat/sample)	dWAIC(bit/sample)	distribution

model_2_1r	hierarchical b	Micro	SCF	RNF	SIS2-3	Stage	1.693953	0.112680	0.16	“log-normal, normal”
model_2_2r	b	Micro	SCF	RNF	SIS2-3	Stage	1.581273	0.000000	0.00	log-normal
model_2_3r	hierarchical b		SCF	RNF	SIS2-3	Stage	1.714071	0.132798	0.19	“log-normal, normal”
model_2_4r	b		SCF	RNF	SIS2-3	Stage	1.596227	0.014954	0.02	log-normal
model_2_5r	hierarchical b						1.798636	0.217363	0.31	“log-normal, normal”
model_2_6r	b						1.676252	0.094979	0.14	log-normal
model_2_e1	b	Micro	SCF	RNF	SIS2-3	Stage	14.984710	13.403437	19.34	exponential
model_2_g1	b	Micro	SCF	RNF	SIS2-3	Stage	1.677251	0.095978	0.14	gamma
model_2_1r_st	hierarchical b	Micro	SCF	RNF	SIS2-3	Stage	1.629536	0.048263	0.07	“log-normal, student_t 4”
model_2_2r_st2	hierarchical b	Micro	SCF	RNF	SIS2-3	Stage	1.650141	0.068868	0.10	“log-normal, student_t 6”
model_2_2r_st3	hierarchical b	Micro	SCF	RNF	SIS2-3	Stage	1.662289	0.081016	0.12	“log-normal, student_t 8”
model_2_2r_st4	hierarchical b	Micro	SCF	RNF	SIS2-3	Stage	1.633246	0.051973	0.07	“log-normal, student_t 2”

Also, we use the Kolmogorov-Sminorv statistic to measure the difference of pairs of posterior distributions.

plots

plot of non-censored data

We have a lot of censored interphase duration data (see the lineage tree plots). First, we visualize the non-censored data:

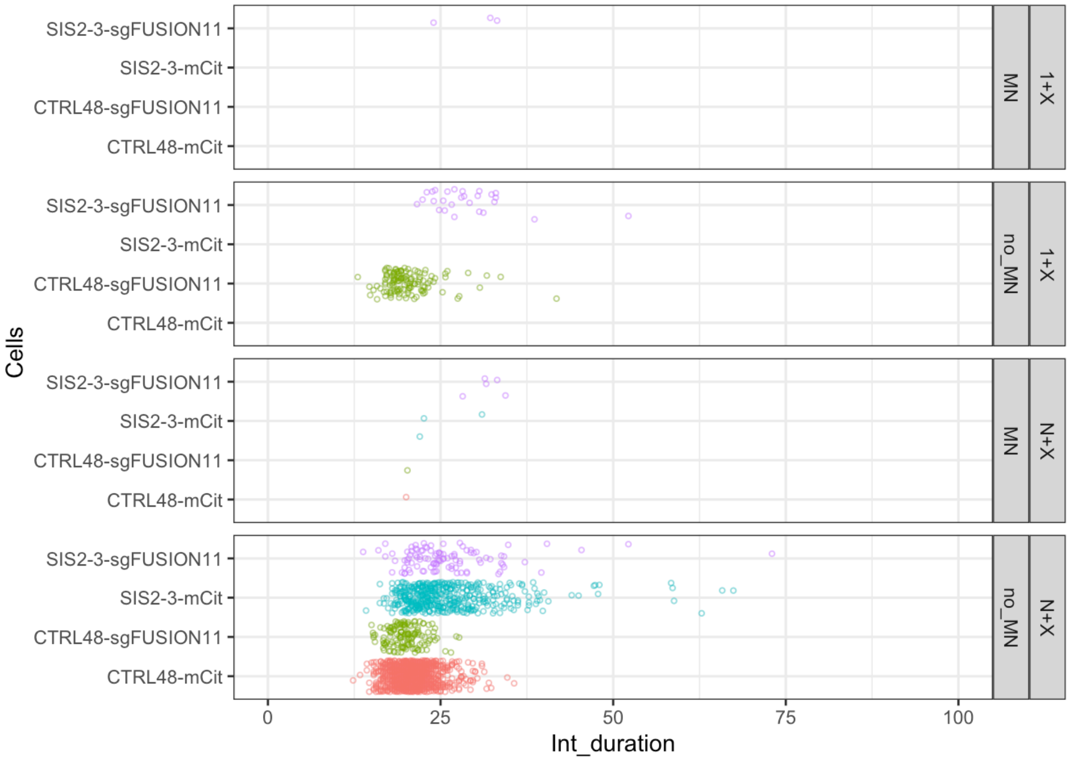

The total number of the cells is 4424. We have many non-censored data for no_MN and N+X condition (at the bottom).

plot of censored data

Next, let's visualize the censored data:

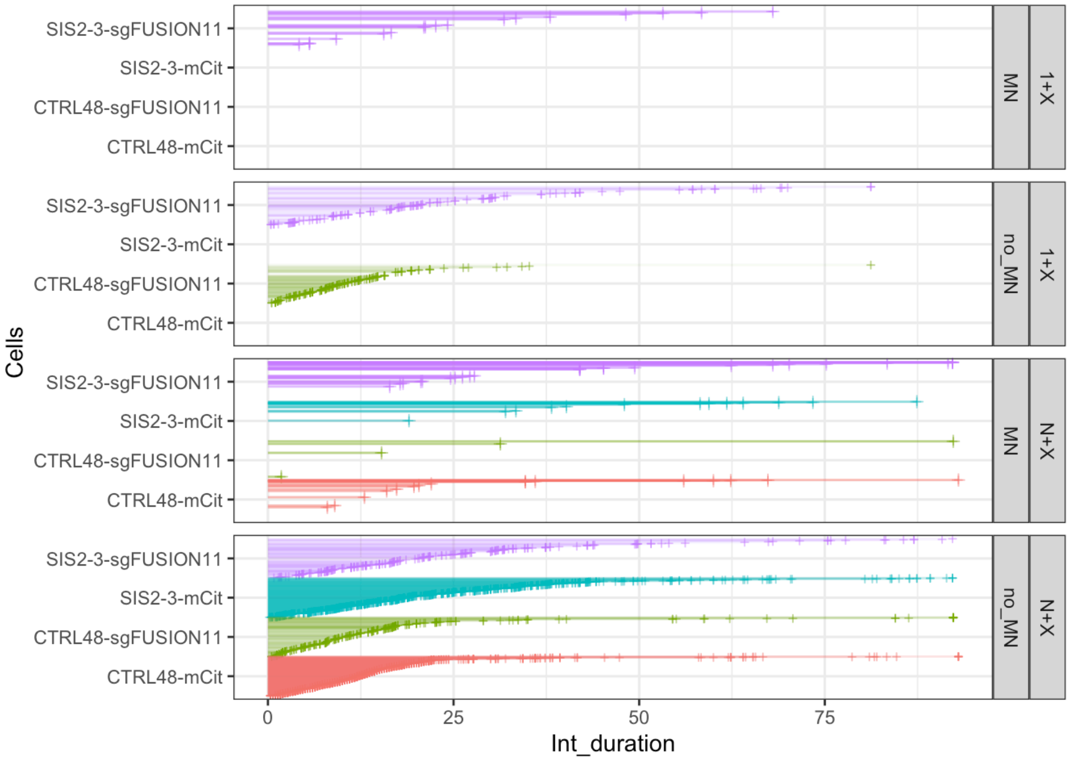

The number of the censored data is 2561. The symbol '+' represents the timing whose observation was censored (time_censored). In other words, the interphase duration of the cell holds: time_censored < interphase_duration < infinity.

combined plot

Let's combine the non-censored and censored plots:

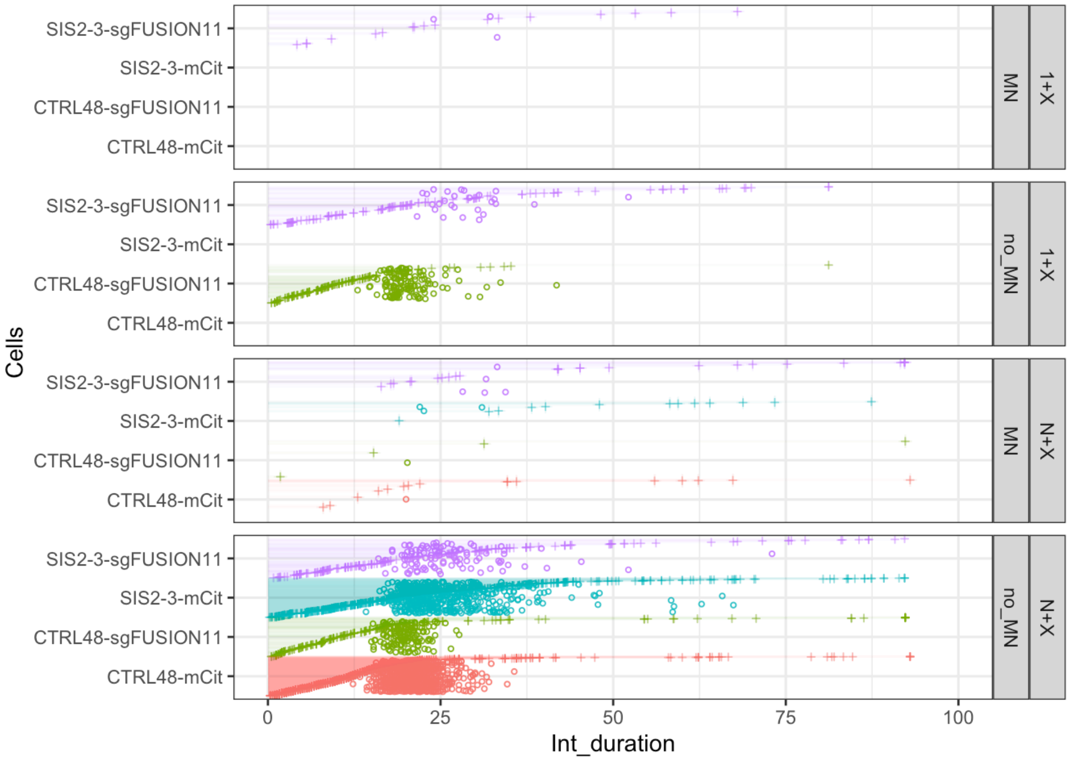

We will construct the models of the probability of the MN formation and the interphase duration using the data.

micronuclei formation probability

The probability $q[n]$ of micronucleus formation can be modeled as a parameter for the Bernoulli distribution:

$$Micronuc[n] \sim \text{Bernoulli}(q[n]), n = 1, \ldots, N_{cell},$$

where $q[n] \in [0, 1]$ and $Micronuc[n] \in \{0, 1\}$.

The probability $q[n]$ can be defined using the inverse logistic function with the linear predictor:

$$q[n] = \text{InvLogit}(b[LineageID[n]] + scf * SCF[n] + rnf * RNF[n] + sis2\_3 * SIS2\_3[n] + stg * Stage[n]),$$
$$n = 1, \ldots, N_{cell},$$

where

$$InvLogit(\alpha) = \frac{1}{1 + \exp(-\alpha)}.$$

The parameters $b[l], l = 1, \ldots N_{lineage}$ can be defined as a random variable subjected to normal distribution with the other parameters, $b0$ and bs:

$$b[l] \sim \text{Normal}(b0, bs), l = 1, \ldots, N_{lineage},$$

where

$$\text{Normal}(y|\mu, \sigma) = \frac{1}{\sqrt{2\pi}\ \sigma} \exp\left(-\frac{1}{2}\left(\frac{y-\mu}{\sigma}\right)^2\right).$$

If we define the $b[l]$ as shown above, we can predict the effect and uncertainty of the lineage individuality as $b0$ and bs based on our definition.

Thus, here $X_n = \{Micronuc_n, LineageID_n, SCF_n, RNF_n, SIS2\_3_n, StageN_n\}$ is the data (observable variables). The parameters (unobservable variables) are $W = \{b0, bs, b_l, scf, rnf, sis2\_3, stg\}$.

One point we should be careful about is that our focus of prediction is about a new cell of a new lineage tree. Therefore, to compare the GLMM with GLM from the predictive point of view, we must marginalize the intermediate parameter ($b[l]$ is the case) when constructing the predictive distribution. The parameters $b[l]$ for each lineage are marginalized out by the numerical integration defined in the function block of the Stan code. The detailed discussion of this part is described in another paper (Harada, Hayashi and Kagaya, 2020).

posterior distributions of the coefficient parameters

We are interested in the quantities W when conditioned by the observed variables: p(w|Data). Let's visualize the distribution through the lens of model 1_1r:

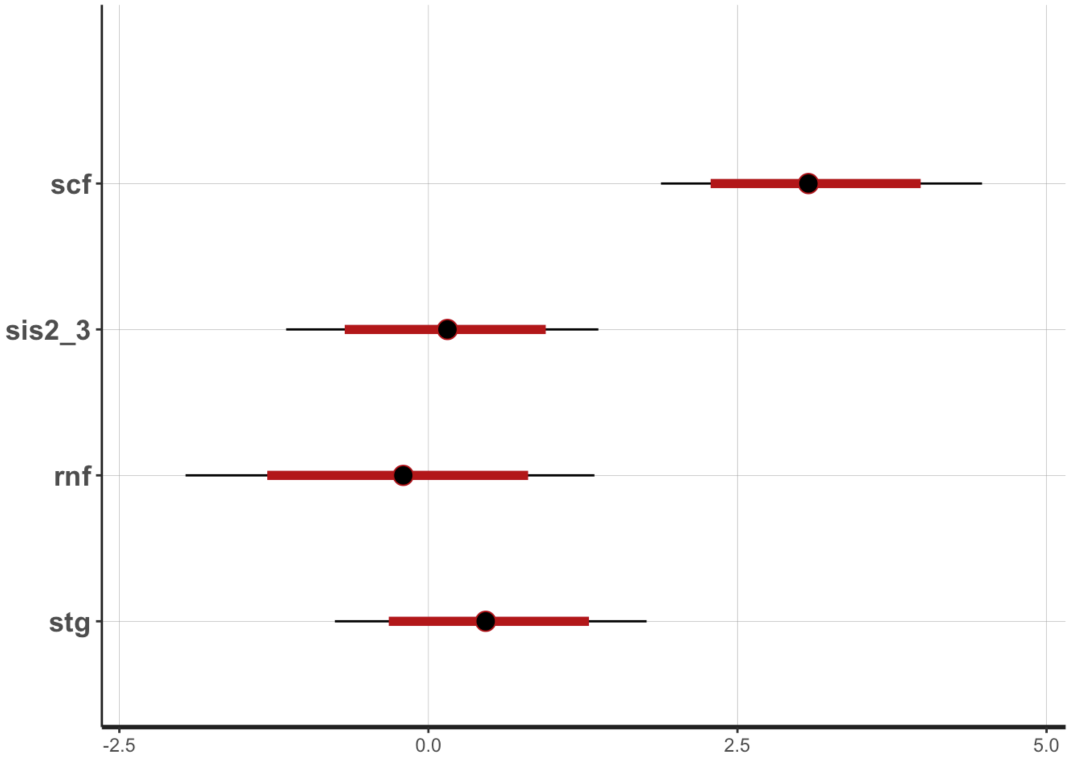

Note that the parameter scf only does not overlap the zero line.

The WAIC for the model 1_1r: 0.1349479.

Next, we remove the assumption of the bias term $b[Lineage[ID]]$. So, we just use a b to all lineages.

The posterior plot of the parameters with the model 1_2r:

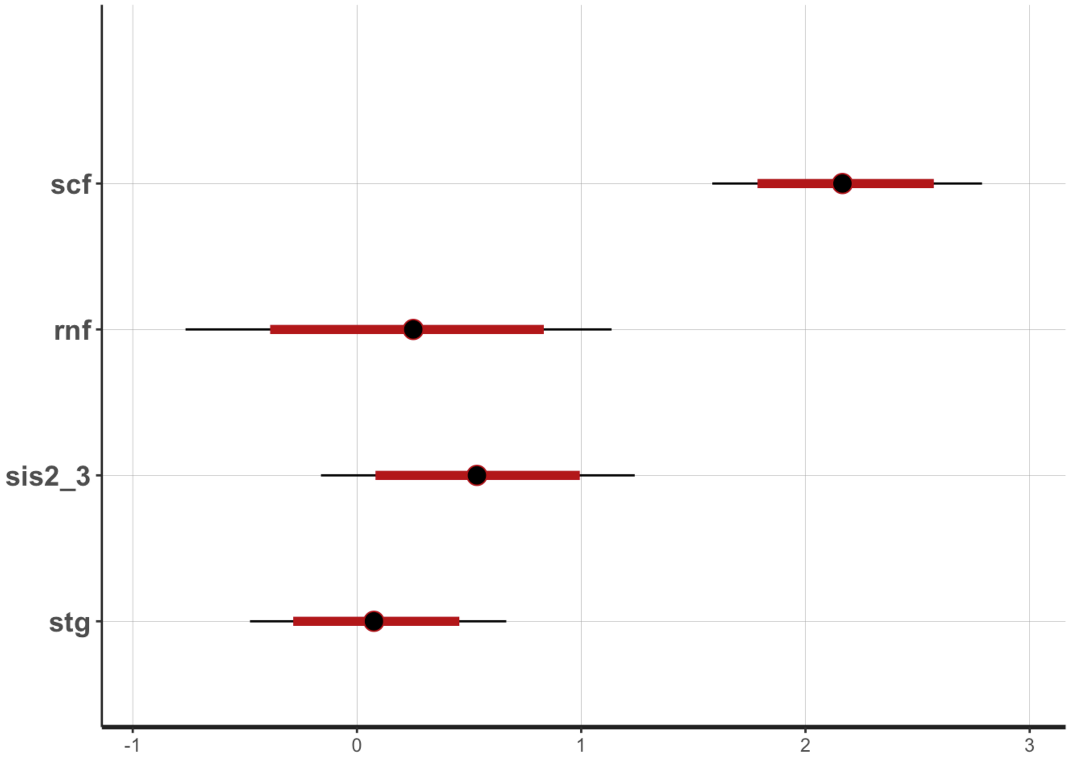

Also, through the lens of this model 1_2, only scf is significantly larger than zero.

The WAIC of the model 1_2r:0.0909744.

mean posterior probability of MN formation with model 1_2

We compute the expected posterior probabilities of MN formation for each experimental conditions:

```
##      [,1]            [,2]
## [1,] "rnf_stageN"    "0.010809550419459"
## [2,] "rng_stage0"    "0.0100493826150542"
## [3,] "sis2_3_stageN" "0.0139014788428874"
## [4,] "sis2_3_stage0" "0.0133707605494688"
## [5,] "scf_stageN"    "0.108068664741875"
## [6,] "scf_stage0"    "0.101683697325042"
## [7,] "ctrl48"        "0.00815191623595166"
```

WAIC for model_1_3r is: 0.0947923.

The posterior distribution of the scf with the model 1_3r:

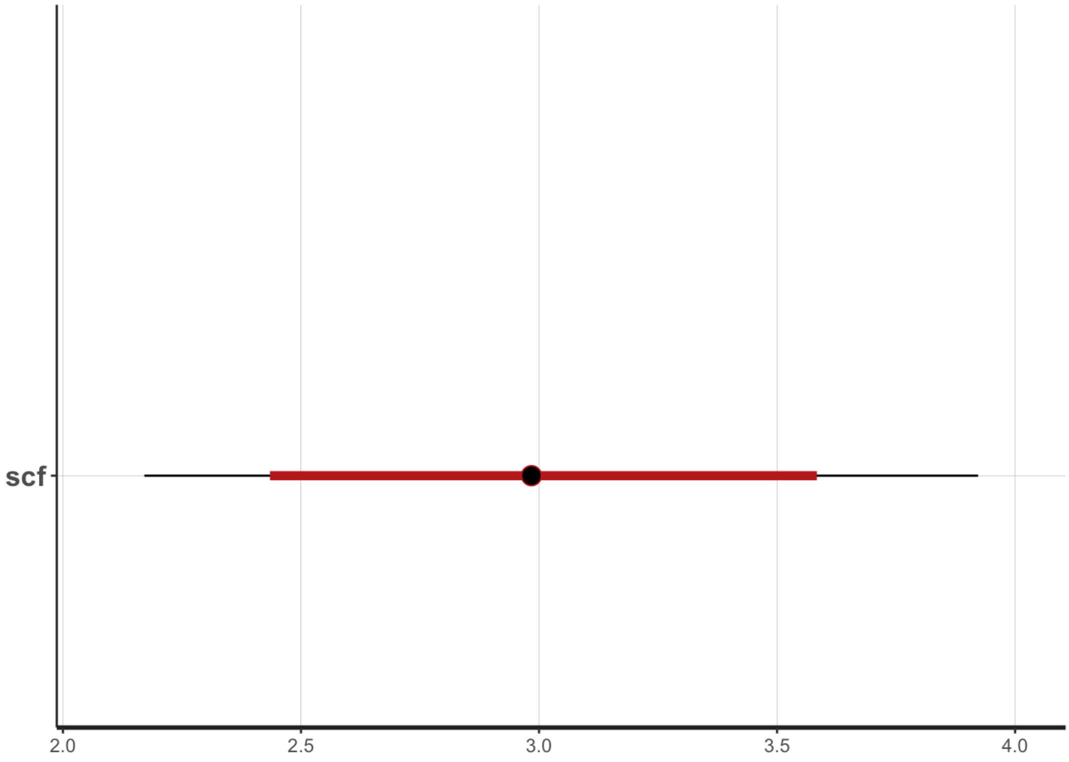

WAIC for the model fit_1_4r is: 0.0905217.

The posterior distribution of the scf with model 1_4r:

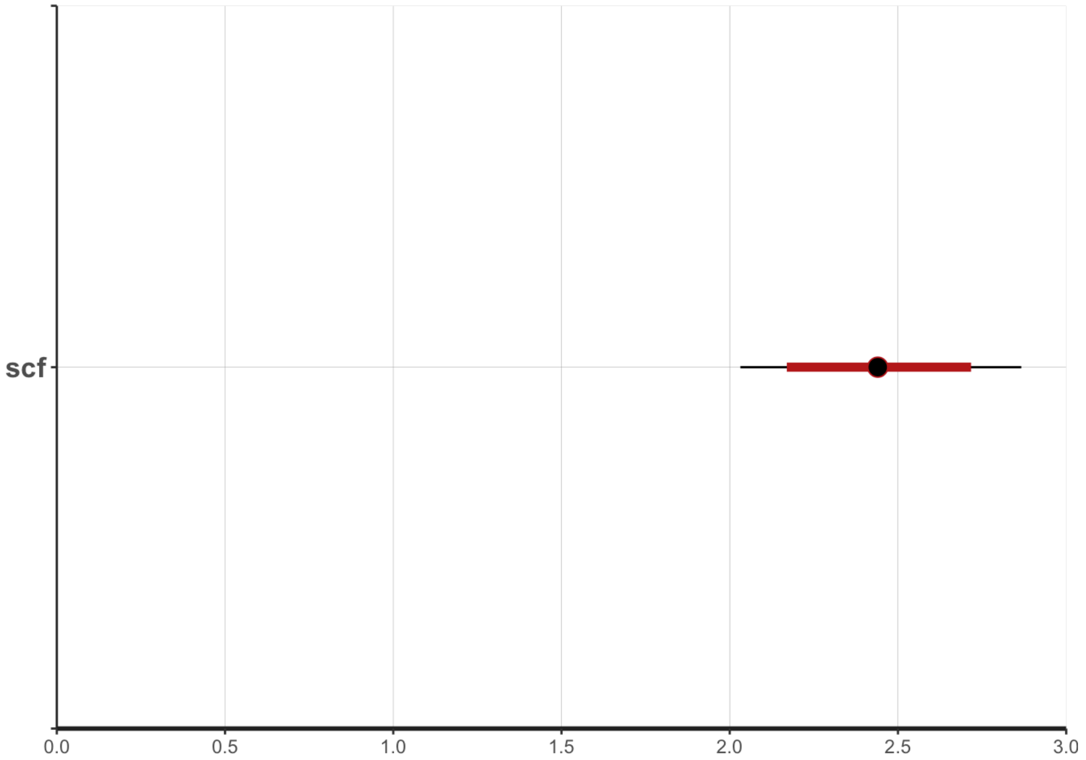

The posterior predictive distribution with the model 1_4r conditioned by if fusion occurred:

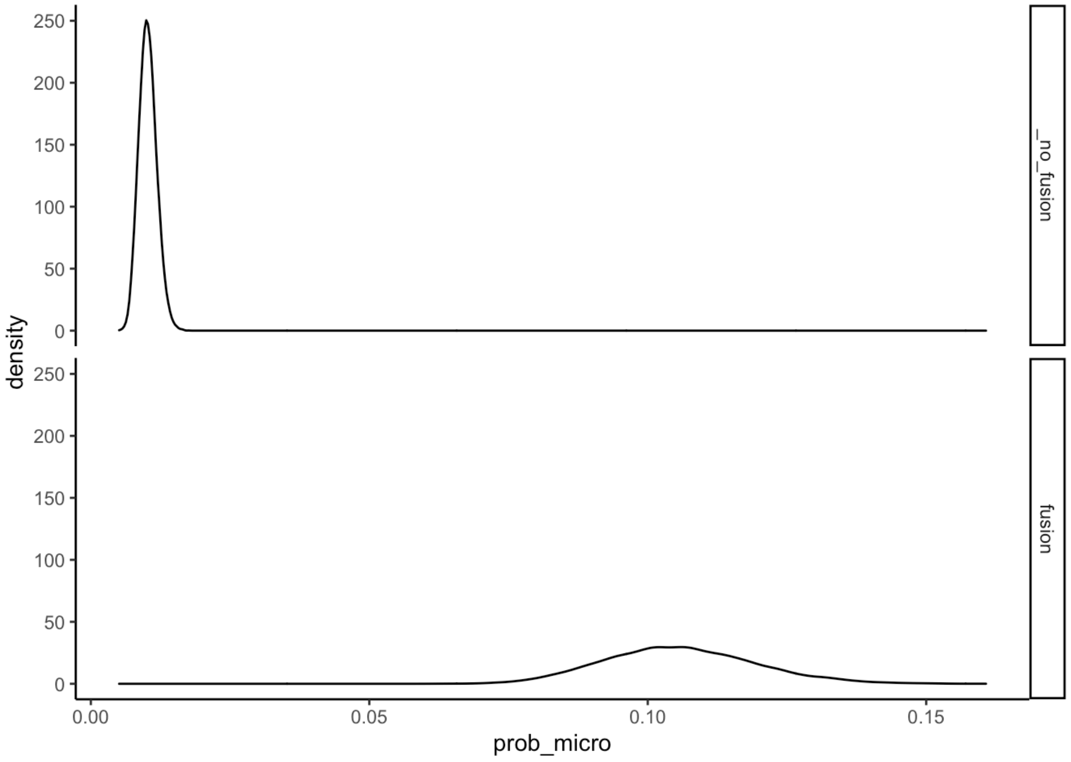

WAIC for the model 1_5r is: 0.1215877.

WAIC for the model 1_6r is: 0.1039501.

We examine the different distribution for the $b[l]$. We tried student t distributions with different degrees of freedom.

WAIC for the model 1_3r_st is: 0.1029526.

WAIC for the model 1_3r_st2 is: 0.1315296.

WAIC for the model 1_3r_st2 is: 0.225825.

interphase duration

The random variable interphase duration is considered to be subject to log-normal distribution in model 2_1.

$$\text{IntDuration}[n] \sim \text{LogNormal}(mu[n], sigma[ExID[n]]), n = 1, \ldots, N_{cell}$$

where

$$\text{LogNormal}(y|\mu, \sigma) = \frac{1}{\sqrt{2\pi}\ \sigma}\frac{1}{y}\ \exp\left(-\frac{1}{2}\left(\frac{\log y - \mu}{\sigma}\right)^2\right).$$

The $sigma[ExID[n]]$ is assigned for each experimental cell group based on the assumption that the instability of the cell cycle is different among the groups.

The parameter $mu[n]$ is linked with the linear predictor as follows:

$$\begin{gathered} mu[n] = micro * Micronuc[n] + scf * SCF[n] + rnf * RNF[n] + \\ sis2\_3 * SIS23[n] + \\ stg * Stage[n] + b[Lineage[n]], \\ n = 1, \ldots, N_{cell} \end{gathered}$$

To infer the causal relationship between some random variables in this type of linear model as the value of the coefficient parameter, we have to close the 'backdoor' which receive flow from upstream confounding factors (Pearl et al., 2016, 'Causal Inference in Statistics'). In other words, we have to condition out the confounding factors to remove the bias. It should be noted that this is another qualitative assessment of the model in addition to the quantitative assessment by WAIC. It is worthy that we interpret the results through the model where this bias is considered even if the WAIC is not a minimum.

Here we focus on the relationship of the random variable $Micronuc$ with the $IntDuration$. The $Micronuc$ is an observational variable, so to infer the degree of causality to $IntDuration$, we have to add the other possibly upstream explanatory variables (scf, rnf, sis2_3, stg). At least we have to add scf based on the results of the previous section.

modeling of censored data

To model the censored data, we used the log-normal complementary cumulative distribution function of Y (Int_duration in our case) given location mu and scale sigma (e.g., see model_2_1r.stan). From the plot shown above, just removing the censored data will generate huge bias to the inference if we do not deal with those data.

results of other models 2_1r ~

WAIC for the model fit_2_1r is: 1.6939534.

The plot of the posterior distribution on the model 2_2r:

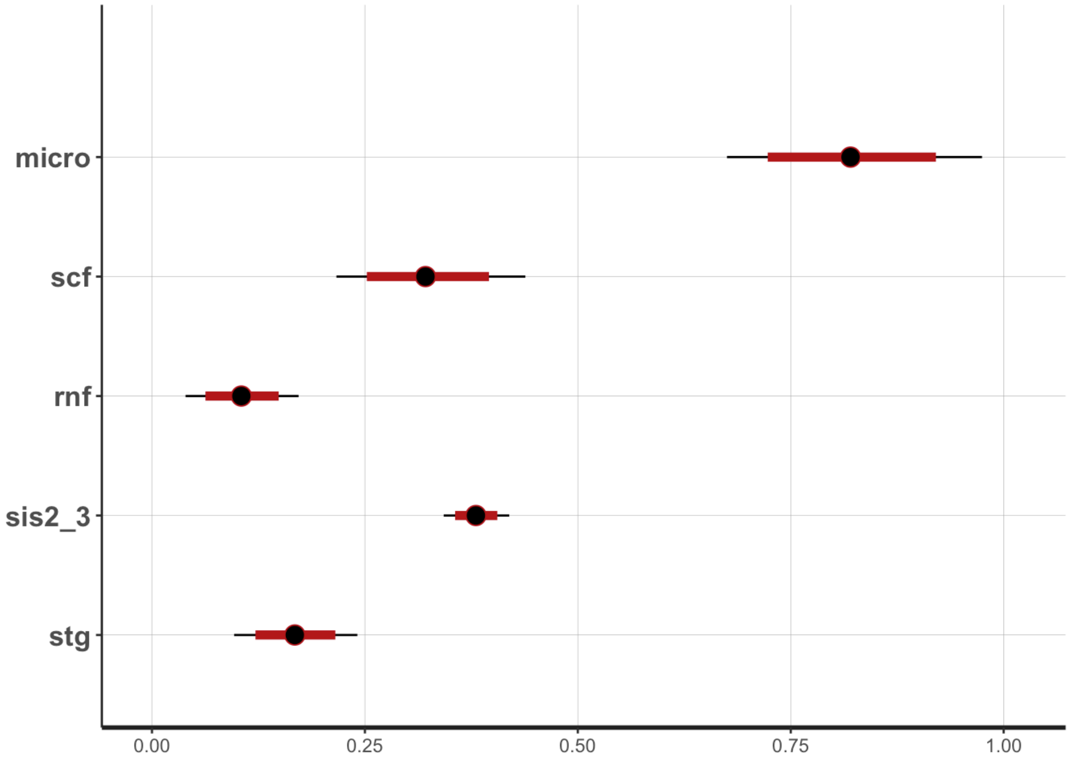

WAIC for the model fit_2_2r is: 1.5812734.

The predictive distribution of the interphase duration with the model 2_2, which performed best among the models:

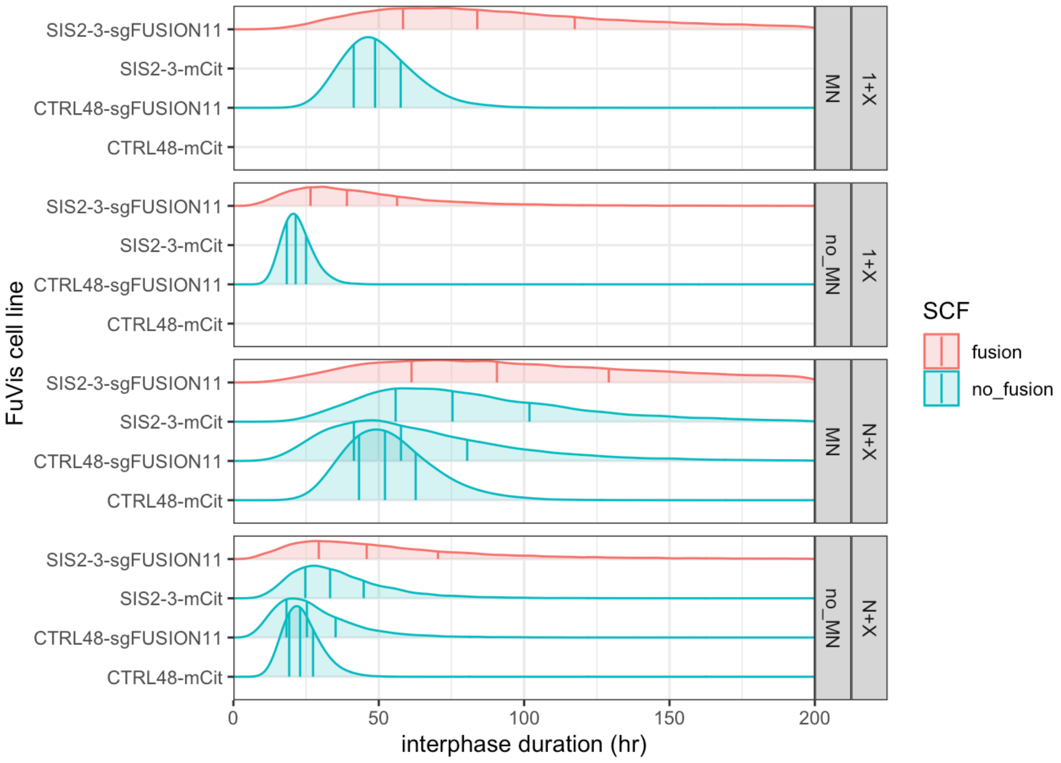

Let's compare the IQR:

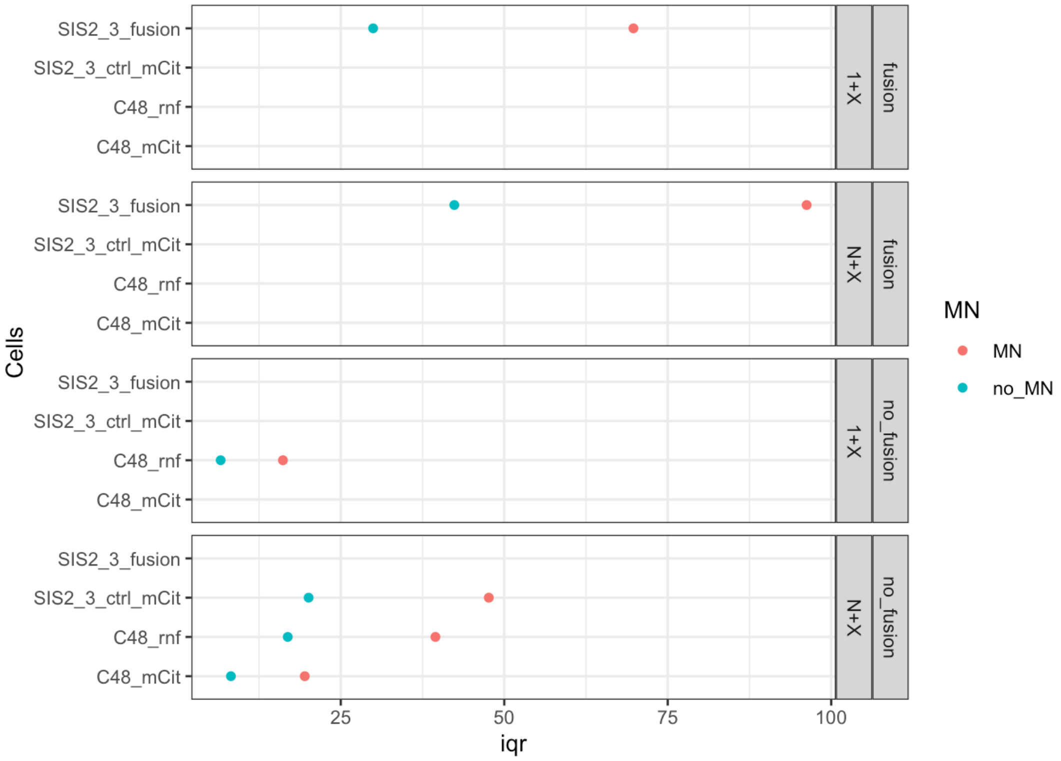

The MN formation prolongs the interquartile range. Thus, the instability of cell cycle appears to increase through MN formation.

model2_1, student_t distribution for individual difference of a lineage tree

WAIC for the model 2_1r_st: 1.6295357.

WAIC for the model 2_1r_st2: 1.6501412.

WAIC for the model 2_1r_st3: 1.6622888.

WAIC for the model 2_1r_st4: 1.6332459.

measuring KS distance of distributions generated from model 2_2

We compute KS distances of the predictive distributions to examine the discrepancies of three pair of the distributions.

comparison of 1+X SIS2_3_fusion_MN & SIS2_3_fusion_no_MN:

```
##
##  One-sample Kolmogorov-Smirnov test
##
## data:  ms2_2$pInt_5_MN
## D = 0.5402, p-value < 2.2e-16
## alternative hypothesis: two-sided
```

comparison of N+X SIS2_3_fusion_MN & SIS2_3_fusion_no_MN

```
##
##  One-sample Kolmogorov-Smirnov test
##
## data:  ms2_2$pInt_6_MN
## D = 0.4659, p-value < 2.2e-16
## alternative hypothesis: two-sided
```

comparison of N+X SIS2_3_ctrl_mCit_MN & SIS2_3_SIS2_3_ctrl_mCit

```
##
##  One-sample Kolmogorov-Smirnov test
##
## data:  ms2_2$pInt_1_MN
## D = 0.64685, p-value < 2.2e-16
## alternative hypothesis: two-sided
```

In either case, the discrepancies were found to be extremely large in the distances.

WAICs for other models:

WAIC for the model 2_3r is: 1.7140714.

WAIC for the model 2_4r is: 1.5962267.

WAIC for the model 2_5r is: 1.7986364.

WAIC for the model 2_6r is: 1.6762522.

WAIC for the model 2_e1 is: 14.9847064.

WAIC for the model 2_g1 is: 1.6772514.

The exponential and gamma distributions were found to be worse than the log-normal distribution.

references

Akaike, Hirotugu. “A new look at the statistical model identification.” IEEE transactions on automatic control 19.6 (1974): 716-723.

Aarts, Emmeke, et al. “Multilevel analysis quantifies variation in the experimental effect while optimizing power and preventing false positives.” BMC neuroscience 16.1 (2015): 94.

Lord, Samuel J., et al. “SuperPlots: Communicating reproducibility and variability in cell biology.” Journal of Cell Biology 219.6 (2020).

Harada, Keita, Naoki Hayashi, and Katsushi Kagaya. “Individual behavioral type captured by a Bayesian model comparison of cap making by sponge crabs.” PeerJ 8 (2020): e9036.

Sakamoto, Yosiyuki, Makio Ishiguro, and Genshiro Kitagawa. “Akaike information criterion statistics.” Dordrecht, The Netherlands: D. Reidel 81 (1986).

Watanabe, Sumio. “Mathematical theory of Bayesian statistics.” (2018).

session info (R, package versions etc.)

```
## R version 3.6.3 (2020-02-29)
## Platform: x86_64-apple-darwin15.6.0 (64-bit)
## Running under: macOS Catalina 10.15.6
##
## Matrix products: default
## BLAS:   /Library/Frameworks/R.framework/Versions/3.6/Resources/lib/libRblas.0.dylib
## LAPACK: /Library/Frameworks/R.framework/Versions/3.6/Resources/lib/libRlapack.dylib
##
## locale:
## [1] en_US.UTF-8/ja_JP.UTF-8/en_US.UTF-8/C/en_US.UTF-8/en_US.UTF-8
##
## attached base packages:
## [1] stats     graphics  grDevices utils     datasets  methods   base
##
## other attached packages:
##  [1] KSgeneral_1.0.0      ggridges_0.5.2       dummies_1.5.6
##  [4] rstan_2.21.2         StanHeaders_2.21.0-6 forcats_0.5.0
##  [7] stringr_1.4.0        dplyr_1.0.2          purrr_0.3.4
## [10] readr_1.3.1          tidyr_1.1.2          tibble_3.0.3
## [13] ggplot2_3.3.2        tidyverse_1.3.0
##
## loaded via a namespace (and not attached):
##  [1] httr_1.4.2         jsonlite_1.7.1     modelr_0.1.8       RcppParallel_5.0.2
##  [5] assertthat_0.2.1   stats4_3.6.3       blob_1.2.1         cellranger_1.1.0
##  [9] yaml_2.2.1         pillar_1.4.6       backports_1.1.10   glue_1.4.2
## [13] digest_0.6.25      rvest_0.3.6        colorspace_1.4-1   htmltools_0.5.0
## [17] plyr_1.8.6         pkgconfig_2.0.3    broom_0.7.0        haven_2.3.1
## [21] scales_1.1.1       processx_3.4.4     generics_0.0.2     farver_2.0.3
## [25] ellipsis_0.3.1     withr_2.2.0        dgof_1.2           cli_2.0.2
## [29] magrittr_1.5       crayon_1.3.4       readxl_1.3.1       evaluate_0.14
## [33] ps_1.3.4           fs_1.5.0           fansi_0.4.1        MASS_7.3-53
## [37] xml2_1.3.2         pkgbuild_1.1.0     tools_3.6.3        loo_2.3.1
## [41] prettyunits_1.1.1  hms_0.5.3          lifecycle_0.2.0    matrixStats_0.56.0
## [45] V8_3.2.0           munsell_0.5.0      reprex_0.3.0       callr_3.4.4
## [49] compiler_3.6.3     rlang_0.4.7        grid_3.6.3         rstudioapi_0.11
## [53] labeling_0.3       rmarkdown_2.3      gtable_0.3.0       codetools_0.2-16
## [57] inline_0.3.16      DBI_1.1.0          curl_4.3           R6_2.4.1
## [61] gridExtra_2.3      lubridate_1.7.9    knitr_1.29         stringi_1.5.3
## [65] parallel_3.6.3     Rcpp_1.0.5         vctrs_0.3.4        dbplyr_1.4.4
## [69] tidyselect_1.1.0   xfun_0.17
```
