# Supplementary material for: Chromosome instability induced by a single defined sister chromatid fusion
Source: Life Sci Alliance. 2020 Oct 26;3(12):e202000911. doi: 10.26508/lsa.202000911 (PMC7652394; doi:10.26508/lsa.202000911)
Supplement: Supplementary file 11 [file LSA-2020-00911_Supplemental_Data_3.docx]

Sequence_S2

pMTH729

gcgcccaatacgcaaaccgcctctccccgcgcgttggccgattcattaatgcagctggcacgacaggtttcccgactggaaagcgggcagtgagcgcaacgcaattaatgtgagttagctcactcattaggcaccccaggctttacactttatgcttccggctcgtatgttgtgtggaattgtgagcggataacaatttcacacaggaaacagctatgaccatgattacgaattgaagactcgagttgtaccctccatcgcctcttccaatctcaagcaattccataacttggaaaacctcaggcaaggacttcctggaatatgtccactgtaacgaccaggttttccagtgtgttatctacaccctgtaacgctgttaggtacataatgtttcagcaatctttgttcttcaccagcactctgagtacatgaaaaaggccaagatgcttcttcagggatgaattttgctactttttaaaggagacttaagaggcacttttggcactctaagtctttcttcaaatgatgaagtttgttacctatttaactcattgctgtgacgcgttttccaattctatgttcccttggtttttgttgtatttttttctgcatgaactctacatcatttactcactctgaacgacagaataaaagaaattggccaccatatcatactcggaaggacaatcatggccatgagacacaaaggactcccagccctgggcccaggcccccctcacgcatgcagccatcgcggcactgtgcctgagtgggccatatacatggtggggacccgatgctgggagacacagctcagggcacaggggccccaagaagccatagctggggaaagctcattcccgacagggctcagctccaacctgaaactagagtcccaccctggggtttccatggtggtggtaaaccaaccacagattttggggatatgactgctccctttgccacgatagcttctcccacgtgcccctggcctgatgaccagaccactagagacgggaggcccgagtcccagggatgggtgggttgcaggcagagctggggctggatggacggtgagtggtgagagctcaaggtgcagaaggggctcctgtcggggactgggttaacagggaccgggacaaatagacggggacttccgagatgagaaagaccttttcgtacaaagtgtttgcatcagtacctcacaatgaaaagaataagataaataacagtacaaaaaagcaatcaccagatcagctcaaggcatcgagcggccgcgtcgagcgcgccaagggagagcccccccccaaagcccccagggatgtaattgcatccctcttccgctagggggcagcagcgagccgcccggggctccgctccggtccggcgctccccccgcatccccgagccggagccggcagcgtgcggggacagcccggcacggggaaggtggcacgcgatcgctttcctctgaacgcttctcgctgctctttgagcctgcagacacctggggggatacggggaaaaagctttaggctgaaagagagatttagaatgacagaatcatagaatggcctgggttgcaaaggagcacagtgctcacccagctccaaccccctgctatgtgcagggtcgccaaccagcagcccaggctgcccagagccacatccagcctggccttgaatgcgcgcgccaagggagagcccccccccaaagcccccagggatgtaattgcatccctcttccgctagggggcagcagcgagccgcccggggctccgctccggtccggcgctccccccgcatccccgagccggagccggcagcgtgcggggacagcccggcacggggaaggtggcacgcgatcgctttcctctgaacgcttctcgctgctctttgagcctgcagacacctggggggatacggggaaaaagctttaggctgaaagagagatttagaatgacagaatcatagaatggcctgggttgcaaaggagcacagtgctcacccagctccaaccccctgctatgtgcagggtcgccaaccagcagcccaggctgcccagagccacatccagcctggccttgaatgcgctcgacgaacaaacgacccaacaccgtgcgttttattctgtctttttattgccgtcgaggctgatcagcgagctcgctgatcagctaggttatttcttcttcttagcctgtccagccttctttgtggcggcaggtcgcttggtacccttatacagctcatccattccaagcgtgattcctgctgctgtgacaaactcaaggagcaccatgtggtcacgtttctcattggggtctttggacagtttggactgatagctcaggtagtgattatcgggcaacagcacaggcccatctcctataggggtattctgctggtagtgatccgccagttgaacagatccatcctcaatgttgtggcgaatcttgaagttcaccttgatcccgttcttctgcttatcggccatgatgtacacgttatggctattgtagttgtattcgagtttatgccccagtatgttgccatcttccttgaagtcgatccctttcagttcgatccgattgaccagtgtgtctccctcaaacttcacctcagctcgtgtcttgtagttgccgtcgtccttgaagaaaatcgtcctctcttgcacataaccttccggcatagcactcttgaagaagtcatgctgcttcatatggtctgggtatctggcaaagcacatcagaccatacccaaaggtagtgaccaaagttggccaaggaactggaagtttgcctgcaaggaaaagaagaagaagtagagttagtgcactaaacaagattcaactaaaacattaaaacaaaaacaatcctttcaacataaccactgatgtcttgttgatagctagtggagtgaacgttggcactacttcacaggagtactccgtagccttatccatgtatgacactcctgtaatacgttattctaccacggcagtcgttcggttgatatcggagtgcttgagcacatacgcgaattggagtatcccttacgcgagatcgttccggtcactccaacgccggacacgttcgctacggatcacccatagagcccaccgcatccccagcatgcctgctattgtcttcccaatcctcccccttgctgtcctgccccaccccaccccccagaatagaatgacacctactcagacaatgcgatgcaatttcctcattttattaggaaaggacagtgggagtggcaccttccagggtcaaggaaggcacgggggaggggcaaacaacagatggctggcaactagaaggcacaggatcgtcgacgaacaaacgacccaacaccgtgcgttttattctgtctttttattgccgtcatagcgcgggttccttccggtattgtctccttccgtgtttcagttagcctttacattactgcagttaaagctttcagaagaactcgtcaagaaggcgatagaaggcgatgcgctgcgaatcgggagcggcgataccgtaaagcacgaggaagcggtcagcccattcgccgccaagctcttcagcaatatcacgggtagccaacgctatgtcctgatagcggtccgccacacccagccggccacagtcgatgaatccagaaaagcggccattttccaccatgatattcggcaagcaggcatcgccatgggtcacgacgagatcctcgccgtcgggcatgcgcgccttgagcctggcgaacagttcggctggcgcgagcccctgatgctcttcgtccagatcatcctgatcgacaagaccggcttccatccgagtacgtgctcgctcgatgcgatgtttcgcttggtggtcgaatgggcaggtagccggatcaagcgtatgcagccgccgcattgcatcagccatgatggatactttctcggcaggagcaaggtgagatgacaggagatcctgccccggcacttcgcccaatagcagccagtcccttcccgcttcagtgacaacgtcgagcacagctgcgcaaggaacgcccgtcgtggccagccacgatagccgcgctgcctcgtcctgcagttcattcagggcaccggacaggtcggtcttgacaaaaagaaccgggcgcccctgcgctgacagccggaacacggcggcatcagagcagccgattgtctgttgtgcccagtcatagccgaatagcctctccacccaagcggccggagaacctgcgtgcaatccatcttgttcaatcatcggtccaggattctcttcgacatctccggcttgtttcagcagagagaagtttgttgcccctgcaagaagaagaagaagaggtagagtcagtgcactaaacaagattccactatagggataacagggtaatggatccgtagcgaacgtgtccggcgttggagtgaccggaacgatctcgcgtaagggatactccaattcgcgtatgtgctcaagcactccgatatcaaccgaacgactgccgtggtagaataacgtattacaggagtgtcatacatggataaggctacggagtactcctgtgaagtagtgccaacgttcactccactagtgatgcttcgtgtatcgatccatgtaatgcgttcgacggagtgtcgtgaagtgcattcgatctggagtgaattcataacttcgtataatgtatgctatacgaagttataatccaaatcgcttttctcaaattacttacctgtggtacagatgaacttcagagtgagtttgccataggtggcatcgccctctccttcgcctgaaacgctaaatttgtggccattcacgtcaccgtccagttctacgagaatggggactacgccggtaaacaactcttcacctttagacaccatggtggcagcgctctagaaccggtcctgtgttctggcggcaaacccgttgcgaaaaagaacgttcacggcgactactgcacttatatacggttctcccccaccctcgggaaaaaggcggagccagtacacgacatcactttcccagtttaccccgcgccaccttctctaggcaccggatcaattgccgacccctccccccaacttctcggggactgtgggcgatgtgcgctctgccctcgacatagctagcatcggtaacttgtttattgcagcttataatggttacaaataaagcaatagcatcacaaatttcacaaataaagcatttttttcactgcattctagttgtggtttgtccaaactcatcaatgtatcttactgcatctcgagcgcattcaaggccaggctggatgtggctctgggcagcctgggctgctggttggcgaccctgcacatagcagggggttggagctgggtgagcactgtgctcctttgcaacccaggccattctatgattctgtcattctaaatctctctttcagcctaaagctttttccccgtatccccccaggtgtctgcaggctcaaagagcagcgagaagcgttcagaggaaagcgatcgcgtgccaccttccccgtgccgggctgtccccgcacgctgccggctccggctcggggatgcggggggagcgccggaccggagcggagccccgggcggctcgctgctgccccctagcggaagagggatgcaattacatccctgggggctttgggggggggctctcccttggcgcgcgcattcaaggccaggctggatgtggctctgggcagcctgggctgctggttggcgaccctgcacatagcagggggttggagctgggtgagcactgtgctcctttgcaacccaggccattctatgattctgtcattctaaatctctctttcagcctaaagctttttccccgtatccccccaggtgtctgcaggctcaaagagcagcgagaagcgttcagaggaaagcgatcgcgtgccaccttccccgtgccgggctgtccccgcacgctgccggctccggctcggggatgcggggggagcgccggaccggagcggagccccgggcggctcgctgctgccccctagcggaagagggatgcaattacatccctgggggctttgggggggggctctcccttggcgcgctcgagatgcaggcggccgcagcttggcactggccgtcgttttacaacgtcgtgactgggaaaaccctggcgttacccaacttaatcgccttgcagcacatccccctttcgccagctggcgtaatagcgaagaggcccgcaccgatcgcccttcccaacagttgcgcagcctgaatggcgaatggcgcgtagggaagttggaagacatatctgtgtggcccatagagagtagatcccaaagacagaaggcccaggtccctaaatccccacaggggaactgtgttacagaccaggagctcatgtacagggctgtcccagggcccctaaattccagaagggaactgggttagagaccaggggctgatgtaacgggctgtccctggtcccctaaatccccacaggggaactgggttagagatgaggagctcattttccgggctgtccaggtcccctaaatcccagatgggaactgggttatcgaccaggtgctcctctaggggttgtctcagggtcctagtgtgtctggaattggtgggttcttggtctcactgacttcaagaatgaagacgcggaacctcgcggtgagtgttacagttcttaaaggtggcatgtccggagtttgtttcttctgatgttcagatgtgttctgagtttcttctttctggtggggttgtggtctcactggctcaggagtgaagctgcagacctttgcggtgagtgtcacagctcagaaaggcagtgtggacccaaagagtgagcagtagcaagatttattgcaaagagtgaaagaacgaagcttccacagtatggaaagggaccccattgggttgccactgctggctcaggcagtctgcttttattctctaatgtcaggtggcacttttcggggaaatgtgcgcggaacccctatttgtttatttttctaaatacattcaaatatgtatccgctcatgagacaataaccctgataaatgcttcaataatattgaaaaaggaagagtatgagtattcaacatttccgtgtcgcccttattcccttttttgcggcattttgccttcctgtttttgctcacccagaaacgctggtgaaagtaaaagatgctgaagatcagttgggtgcacgagtgggttacatcgaactggatctcaacagcggtaagatccttgagagttttcgccccgaagaacgttttccaatgatgagcacttttaaagttctgctatgtggcgcggtattatcccgtattgacgccgggcaagagcaactcggtcgccgcatacactattctcagaatgacttggttgagtactcaccagtcacagaaaagcatcttacggatggcatgacagtaagagaattatgcagtgctgccataaccatgagtgataacactgcggccaacttacttctgacaacgatcggaggaccgaaggagctaaccgcttttttgcacaacatgggggatcatgtaactcgccttgatcgttgggaaccggagctgaatgaagccataccaaacgacgagcgtgacaccacgatgcctgtagcaatggcaacaacgttgcgcaaactattaactggcgaactacttactctagcttcccggcaacaattaatagactggatggaggcggataaagttgcaggaccacttctgcgctcggcccttccggctggctggtttattgctgataaatctggagccggtgagcgtgggtctcgcggtatcattgcagcactggggccagatggtaagccctcccgtatcgtagttatctacacgacggggagtcaggcaactatggatgaacgaaatagacagatcgctgagataggtgcctcactgattaagcattggtaactgtcagaccaagtttactcatatatactttagattgatttaaaacttcatttttaatttaaaaggatctaggtgaagatcctttttgataatctcatgaccaaaatcccttaacgtgagttttcgttccactgagcgtcagaccccgtagaaaagatcaaaggatcttcttgagatcctttttttctgcgcgtaatctgctgcttgcaaacaaaaaaaccaccgctaccagcggtggtttgtttgccggatcaagagctaccaactctttttccgaaggtaactggcttcagcagagcgcagataccaaatactgtccttctagtgtagccgtagttaggccaccacttcaagaactctgtagcaccgcctacatacctcgctctgctaatcctgttaccagtggctgctgccagtggcgataagtcgtgtcttaccgggttggactcaagacgatagttaccggataaggcgcagcggtcgggctgaacggggggttcgtgcacacagcccagcttggagcgaacgacctacaccgaactgagatacctacagcgtgagctatgagaaagcgccacgcttcccgaagggagaaaggcggacaggtatccggtaagcggcagggtcggaacaggagagcgcacgagggagcttccagggggaaacgcctggtatctttatagtcctgtcgggtttcgccacctctgacttgagcgtcgatttttgtgatgctcgtcaggggggcggagcctatggaaaaacgccagcaacgcggcctttttacggttcctggccttttgctggccttttgctcacatgttctttcctgcgttatcccctgattctgtggataaccgtattaccgcctttgagtgagctgataccgctcgccgcagccgaacgaccgagcgcagcgagtcagtgagcgaggaagcggaaga
