# Supplementary material for: Chromosome instability induced by a single defined sister chromatid fusion
Source: Life Sci Alliance. 2020 Oct 26;3(12):e202000911. doi: 10.26508/lsa.202000911 (PMC7652394; doi:10.26508/lsa.202000911)
Supplement: Supplementary file 14 [file LSA-2020-00911_Supplemental_Data_1.zip › stat_modeling_r1.html]

Appendix Supplementary Method


# Appendix Supplementary Method

### overview

This document is written to provide supplementary information for the paper entitled ‘Chromosome Instability Induced by a Single Defined Sister Chromatid Fusion’ by K. Kagaya, N. Noma-Takayasu, I. Yamamoto, S. Tashiro, F. Ishikawa, M. T. Hayashi.

The reasons why we chose the approach (Bayesian model evaluation with WAIC) are three folds:

1. to consider a hierarchical structure (lineage tree),
2. to compare the hierarchical model with non-hierarchical model,
3. to minimize the generalization error (maximum likelihood estimation is worse than Bayes).

The approach is based on the mathematically rigorous theory (Watanabe, 2018, Mathematical Theory of Bayesian Statistics; http://watanabe-www.math.dis.titech.ac.jp/users/swatanab/waicwbic\_e.html).

The framework and procedure we took here may not be a conventional and familiar one for the biological community. However, our approach is beginning to be accepted in the papers (Wakita, Kagaya and Aonuma, 2020 J Royal Soc Interface; Harada, Hayashi and Kagaya, 2020 PeerJ).

Currently, WAIC is the best estimator of the generalization error of the statistical models. For the non-regular models including the generalized linear models (GLMMs), WAIC is more powerful than AIC based on maximum likelihood method.

AIC and WAIC are the estimators of the generalization error: the discrepancy in the unknown true distribution that generates data q(x) and the predictive distribution that built using a set of realized data p(x|Data) (see the discussion of the paper, Harada, Hayashi and Kagaya, 2020 PeerJ).

To build p(x|Data), AIC is based on the maximum likelihood method. AIC has been often used for GLM. However, if we want to compare GLM with GLMM, WAIC based on the Bayesian method is better than AIC. GLMM has been widely used because it is appropriate for the clustered or grouped data (Lord, et al., 2020; Arts, et al., 2015).

Our live cell data has tree structure, so we took into account the structure in the models and used WAIC to evaluate the models from the predictive point of view (Akaike, 1974; Sakamoto, Ishiguro & Kitagawa, 1986; Watanabe, 2018).

Here we walk through the two parts of the statistical inferences:

1. probability of micronucleus(MN) formation (9 models)

| **model\_name** | **intercept** | **X1** | **X2** | **X3** | **X4** | **WAIC** | **dWAIC(nat/sample)** | **dWAIC(bit/sample)** | **distribution** | **distribution2** |
| --- | --- | --- | --- | --- | --- | --- | --- | --- | --- | --- |
| model\_1\_1r | hierarchical\_b | SCF | RNF | SIS2-3 | Stage\_N | 0.1349479 | 0.04442622 | 0.064093485 | Bernoulli | normal(b0, bs) |
| model\_1\_2r | b | SCF | RNF | SIS2-4 | Stage\_N | 0.09097437 | 0.00045269 | 0.000653094 | Bernoulli |  |
| model\_1\_3r | hierarchical\_b | SCF |  |  |  | 0.09479229 | 0.00427061 | 0.006161188 | Bernoulli | normal(b0, bs) |
| model\_1\_4r | b | SCF |  |  |  | 0.09052168 | 0 | 0 | Bernoulli |  |
| model\_1\_5r | hierarchical\_b |  |  |  |  | 0.1215877 | 0.03106602 | 0.044818792 | Bernoulli | normal(b0, bs) |
| model\_1\_6r | b |  |  |  |  | 0.1039501 | 0.01342842 | 0.019373114 | Bernoulli |  |
| model\_1\_3r\_st | hierarchical\_b | SCF |  |  |  | 0.1029526 | 0.01243092 | 0.017934026 | Bernoulli | student\_t(4, b0, bs) |
| model\_1\_4r\_st2 | hierarchical\_b | SCF |  |  |  | 0.1315296 | 0.04100792 | 0.059161921 | Bernoulli | student\_t(2, b0, bs) |
| model\_1\_4r\_st3 | hierarchical\_b | SCF |  |  |  | 0.225825 | 0.13530332 | 0.195201423 | Bernoulli | student\_t(1, b0, bs) |

2. impact of the MN formation to the duration of interphase (12 models)

| **model\_name** | **intercept** | **X1** | **X2** | **X3** | **X4** | **X5** | **WAIC** | **dWAIC(nat/sample)** | **dWAIC(bit/sample)** | **distribution** | **distribution2** |
| --- | --- | --- | --- | --- | --- | --- | --- | --- | --- | --- | --- |
| model\_2\_1r | hierarchical b | Micro | SCF | RNF | SIS2-3 | Stage | 1.693953 | 0.112680 | 0.16 | log-normal | normal(b0, bs) |
| model\_2\_2r | b | Micro | SCF | RNF | SIS2-3 | Stage | 1.581273 | 0.000000 | 0.00 | log-normal |  |
| model\_2\_3r | hierarchical b |  | SCF | RNF | SIS2-3 | Stage | 1.714071 | 0.132798 | 0.19 | log-normal | normal(b0, bs) |
| model\_2\_4r | b |  | SCF | RNF | SIS2-3 | Stage | 1.596227 | 0.014954 | 0.02 | log-normal |  |
| model\_2\_5r | hierarchical b |  |  |  |  |  | 1.798636 | 0.217363 | 0.31 | log-normal | normal(b0, bs) |
| model\_2\_6r | b |  |  |  |  |  | 1.676252 | 0.094979 | 0.14 | log-normal |  |
| model\_2\_e1 | b | Micro | SCF | RNF | SIS2-3 | Stage | 14.984710 | 13.403437 | 19.34 | exponential |  |
| model\_2\_g1 | b | Micro | SCF | RNF | SIS2-3 | Stage | 1.677251 | 0.095978 | 0.14 | gamma |  |
| model\_2\_1r\_st | hierarchical b | Micro | SCF | RNF | SIS2-3 | Stage | 1.629536 | 0.048263 | 0.07 | log-normal | student\_t(4, b0, bs) |
| model\_2\_2r\_st2 | hierarchical b | Micro | SCF | RNF | SIS2-3 | Stage | 1.650141 | 0.068868 | 0.10 | log-normal | student\_t(6, b0, bs) |
| model\_2\_2r\_st3 | hierarchical b | Micro | SCF | RNF | SIS2-4 | Stage | 1.662289 | 0.081016 | 0.12 | log-normal | student\_t(8, b0, bs) |
| model\_2\_2r\_st4 | hierarchical b | Micro | SCF | RNF | SIS2-5 | Stage | 1.633246 | 0.051973 | 0.07 | log-normal | student\_t(2, b0, bs) |

Also, we use the Kolmogorov-Sminorv statistic to measure the difference of pairs of posterior distributions.

### plots

#### plot of non-censored data

We have a lot of censored interphase duration data (see the lineage tree plots). First, we visualize the non-censored data:

The total number of the cells is 4424. We have many non-censored data for no\_MN and N+X condition (at the bottom).

#### plot of censored data

Next, let’s visualize the censored data:

The number of the censored data is 2561. The symbol ‘+’ represents the timing whose observation was censored (time\_censored). In other words, the interphase duration of the cell holds: time\_censored < interphase\_duration < infinity.

#### combined plot

Let’s combine the non-censored and censored plots:

We will construct the models of the probability of the MN formation and the interphase duration using the data.

### micronuclei formation probability

The probability \(q[n]\) of micronucleus formation can be modeled as a parameter for the Bernoulli distribution:

\[ Micronuc[n] \sim Bernoulli(q[n]), n=1,...,N\_{cell}. \]

The probability \(q[n]\) can be defined using the inverse logistic function with the linear predictor:

\[ q[n] = InvLogit(b[LineageID[n]] +
scf\*SCF[n] +
rnf\*RNF[n] +
sis2\\_3\*SIS2\\_3[n] +
stg\*Stage[n]), \\
n=1,...,N\_{cell}.
\]

The parameters \(b[l], l=1,...N\_{lineage}\) can be defined as a random variable subjected to normal distribution with the other parameters, \(b0\) and \(bs\):

\[
b[l] \sim Normal(b0, bs), l=1,...,N\_{lineage}.
\]

If we define the \(b[l]\) as shown above, we can predict the effect and uncertainty of the lineage individuality as \(b0\) and \(bs\) based on our definition.

Thus, here \(X\_n=\{Micronuc\_n, LineageID\_n, SCF\_n, RNF\_n, SIS2\\_3\_n,StageN\_n\}\) is the data (observable variables). The parameters (unobservable variables) are \(W=\{b0, bs, b\_l, scf, rnf, sis2\\_3, stg\}\).

One point we should be careful about is that our focus of prediction is about a new cell of a new lineage tree. Therefore, to compare the GLMM with GLM from the predictive point of view, we must marginalize the intermediate parameter (\(b[l]\) is the case) when constructing the predictive distribution. The parameters \(b[l]\) for each lineage are marginalized out by the numerical integration defined in the function block of the Stan code. The detailed discussion of this part is described in another paper (Harada, Hayashi and Kagaya, 2020).

#### posterior distributions of the coefficient parameters

We are interested in the quantities \(W\) when conditioned by the observed variables: p(w|Data). Let’s visualize the distribution through the lens of model 1\_1r:

Note that the parameter \(scf\) only does not overlap the zero line.

The WAIC for the model 1\_1r: 0.1349479.

Next, we remove the assumption of the bias term \(b[Lineage[ID]]\). So, we just use a \(b\) to all lineages.

The posterior plot of the parameters with the model 1\_2r:

Also, through the lens of this model 1\_2, only \(scf\) is significantly larger than zero.

The WAIC of the model 1\_2r:0.0909744.

#### mean posterior probability of MN formation with model 1\_2

We compute the expected posterior probabilities of MN formation for each experimental conditions:

```
##      [,1]            [,2]                 
## [1,] "rnf_stageN"    "0.010809550419459"  
## [2,] "rng_stage0"    "0.0100493826150542" 
## [3,] "sis2_3_stageN" "0.0139014788428874" 
## [4,] "sis2_3_stage0" "0.0133707605494688" 
## [5,] "scf_stageN"    "0.108068664741875"  
## [6,] "scf_stage0"    "0.101683697325042"  
## [7,] "ctrl48"        "0.00815191623595166"
```

WAIC for model\_1\_3r is: 0.0947923.

The posterior distribution of the \(scf\) with the model 1\_3r:

WAIC for the model fit\_1\_4r is: 0.0905217.

The posterior distribution of the \(scf\) with model 1\_4r:

The posterior predictive distribution with the model 1\_4r conditioned by if fusion occurred:

WAIC for the model 1\_5r is: 0.1215877.

WAIC for the model 1\_6r is: 0.1039501.

We examine the different distribution for the \(b[l]\). We tried student t distributions with different degrees of freedom.

WAIC for the model 1\_3r\_st is: 0.1029526.

WAIC for the model 1\_3r\_st2 is: 0.1315296.

WAIC for the model 1\_3r\_st2 is: 0.225825.

### interphase duration

The random variable interphase duration is considered to be subject to log-normal distribution in model 2\_1.

\[
IntDuration[n] \sim LogNormal(mu[n], sigma[ExID[n]]), n=1,...,N\_{cell}
\]

The \(sigma[ExID[n]]\) is assigned for each experimental cell group based on the assumption that the instability of the cell cycle is different among the groups.

The parameter \(mu[n]\) is linked with the linear predictor as follows:

\[
mu[n] = micro\*Micronuc[n] +
scf\*SCF[n] +
rnf\*RNF[n] + \\
sis2\\_3\*SIS23[n] + \\
stg\*Stage[n] +
b[Lineage[n]],\\
n=1,...,N\_{cell}
\]

To infer the causal relationship between some random variables in this type of linear model as the value of the coefficient parameter, we have to close the ‘backdoor’ which receive flow from upstream confounding factors (Pearl et al., 2016, ‘Causal Inference in Statistics’). In other words, we have to condition out the confounding factors to remove the bias. It should be noted that this is another qualitative assessment of the model in addition to the quantitative assessment by WAIC. It is worthy that we interpret the results through the model where this bias is considered even if the WAIC is not a minimum.

Here we focus on the relationship of the random variable \(Micronuc\) with the \(IntDuration\). The \(Micronuc\) is an observational variable, so to infer the degree of causality to \(IntDuration\), we have to add the other possibly upstream explanatory variables (scf, rnf, sis2\_3, stg). At least we have to add \(scf\) based on the results of the previous section.

#### modeling of censored data

To model the censored data, we used the log-normal complementary cumulative distribution function of Y (Int\_duration in our case) given location mu and scale sigma (e.g., see model\_2\_1r.stan). From the plot shown above, just removing the censored data will generate huge bias to the inference if we do not deal with those data.

#### results of other models 2\_1r ~

WAIC for the model fit\_2\_1r is: 1.6939534.

The plot of the posterior distribution on the model 2\_2r:

WAIC for the model fit\_2\_2r is: 1.5812734.

The predictive distribution of the interphase duration with the model 2\_2, which performed best among the models:

Let’s compare the IQR:

The MN formation prolongs the interquartile range. Thus, the instability of cell cycle appears to increase through MN formation.

#### model2\_1, student\_t distribution for individual difference of a lineage tree

WAIC for the model 2\_1r\_st: 1.6295357.

WAIC for the model 2\_1r\_st2: 1.6501412.

WAIC for the model 2\_1r\_st3: 1.6622888.

WAIC for the model 2\_1r\_st4: 1.6332459.

### measuring KS distance of distributions generated from model 2\_2

We compute KS distances of the predictive distributions to examine the discrepancies of three pair of the distributions.

comparison of 1+X SIS2\_3\_fusion\_MN & SIS2\_3\_fusion\_no\_MN:

```
## 
##  One-sample Kolmogorov-Smirnov test
## 
## data:  ms2_2$pInt_5_MN
## D = 0.5402, p-value < 2.2e-16
## alternative hypothesis: two-sided
```

comparison of N+X SIS2\_3\_fusion\_MN & SIS2\_3\_fusion\_no\_MN

```
## 
##  One-sample Kolmogorov-Smirnov test
## 
## data:  ms2_2$pInt_6_MN
## D = 0.4659, p-value < 2.2e-16
## alternative hypothesis: two-sided
```

comparison of N+X SIS2\_3\_ctrl\_mCit\_MN & SIS2\_3\_SIS2\_3\_ctrl\_mCit

```
## 
##  One-sample Kolmogorov-Smirnov test
## 
## data:  ms2_2$pInt_1_MN
## D = 0.64685, p-value < 2.2e-16
## alternative hypothesis: two-sided
```

In either case, the discrepancies were found to be extremely large in the distances.

WAICs for other models:

WAIC for the model 2\_3r is: 1.7140714.

WAIC for the model 2\_4r is: 1.5962267.

WAIC for the model 2\_5r is: 1.7986364.

WAIC for the model 2\_6r is: 1.6762522.

WAIC for the model 2\_e1 is: 14.9847064.

WAIC for the model 2\_g1 is: 1.6772514.

The exponential and gamma distributions were found to be worse than the log-normal distribution.

### references

Akaike, Hirotugu. “A new look at the statistical model identification.” IEEE transactions on automatic control 19.6 (1974): 716-723.

Aarts, Emmeke, et al. “Multilevel analysis quantifies variation in the experimental effect while optimizing power and preventing false positives.” BMC neuroscience 16.1 (2015): 94.

Lord, Samuel J., et al. “SuperPlots: Communicating reproducibility and variability in cell biology.” Journal of Cell Biology 219.6 (2020).

Harada, Keita, Naoki Hayashi, and Katsushi Kagaya. “Individual behavioral type captured by a Bayesian model comparison of cap making by sponge crabs.” PeerJ 8 (2020): e9036.

Sakamoto, Yosiyuki, Makio Ishiguro, and Genshiro Kitagawa. “Akaike information criterion statistics.” Dordrecht, The Netherlands: D. Reidel 81 (1986).

Watanabe, Sumio. “Mathematical theory of Bayesian statistics.” (2018).

### session info (R, package versions etc.)

```
## R version 3.6.3 (2020-02-29)
## Platform: x86_64-pc-linux-gnu (64-bit)
## Running under: Ubuntu 18.04.4 LTS
## 
## Matrix products: default
## BLAS:   /usr/lib/x86_64-linux-gnu/blas/libblas.so.3.7.1
## LAPACK: /usr/lib/x86_64-linux-gnu/lapack/liblapack.so.3.7.1
## 
## locale:
##  [1] LC_CTYPE=en_US.UTF-8       LC_NUMERIC=C              
##  [3] LC_TIME=en_US.UTF-8        LC_COLLATE=en_US.UTF-8    
##  [5] LC_MONETARY=en_US.UTF-8    LC_MESSAGES=en_US.UTF-8   
##  [7] LC_PAPER=en_US.UTF-8       LC_NAME=C                 
##  [9] LC_ADDRESS=C               LC_TELEPHONE=C            
## [11] LC_MEASUREMENT=en_US.UTF-8 LC_IDENTIFICATION=C       
## 
## attached base packages:
## [1] stats     graphics  grDevices utils     datasets  methods   base     
## 
## other attached packages:
##  [1] KSgeneral_0.1.2    ggridges_0.5.1     dummies_1.5.6      rstan_2.19.3      
##  [5] StanHeaders_2.19.0 forcats_0.4.0      stringr_1.4.0      dplyr_0.8.3       
##  [9] purrr_0.3.3        readr_1.3.1        tidyr_1.0.0        tibble_2.1.3      
## [13] ggplot2_3.2.1      tidyverse_1.3.0   
## 
## loaded via a namespace (and not attached):
##  [1] httr_1.4.1         jsonlite_1.6       modelr_0.1.5       assertthat_0.2.1  
##  [5] stats4_3.6.3       cellranger_1.1.0   yaml_2.2.0         pillar_1.4.3      
##  [9] backports_1.1.5    lattice_0.20-40    glue_1.3.1         digest_0.6.23     
## [13] rvest_0.3.5        colorspace_1.4-1   htmltools_0.4.0    plyr_1.8.5        
## [17] pkgconfig_2.0.3    broom_0.5.3        haven_2.2.0        scales_1.1.0      
## [21] processx_3.4.1     generics_0.0.2     farver_2.0.1       withr_2.1.2       
## [25] dgof_1.2           lazyeval_0.2.2     cli_2.0.0          magrittr_1.5      
## [29] crayon_1.3.4       readxl_1.3.1       evaluate_0.14      ps_1.3.0          
## [33] fs_1.3.1           fansi_0.4.0        nlme_3.1-144       MASS_7.3-51.5     
## [37] xml2_1.2.2         pkgbuild_1.0.6     tools_3.6.3        loo_2.2.0         
## [41] prettyunits_1.0.2  hms_0.5.2          lifecycle_0.1.0    matrixStats_0.55.0
## [45] munsell_0.5.0      reprex_0.3.0       callr_3.4.0        compiler_3.6.3    
## [49] rlang_0.4.6        grid_3.6.3         rstudioapi_0.10    labeling_0.3      
## [53] rmarkdown_2.0      gtable_0.3.0       inline_0.3.15      DBI_1.1.0         
## [57] reshape2_1.4.3     R6_2.4.1           gridExtra_2.3      lubridate_1.7.4   
## [61] knitr_1.26         zeallot_0.1.0      stringi_1.4.3      parallel_3.6.3    
## [65] Rcpp_1.0.3         vctrs_0.2.1        dbplyr_1.4.2       tidyselect_0.2.5  
## [69] xfun_0.11
```
